# Genetic divergence and evidence of human‐mediated translocation of two‐fingered sloths (C*holoepus hoffmanni*) in Costa Rica

**DOI:** 10.1111/eva.13036

**Published:** 2020-06-26

**Authors:** Rebecca N. Cliffe, Chloe V. Robinson, Benjamin A. Whittaker, Sarah J. Kennedy, Judy A. Avey‐Arroyo, Sofia Consuegra, Rory P. Wilson

**Affiliations:** ^1^ Biosciences, College of Science Swansea University Wales UK; ^2^ The Sloth Sanctuary of Costa Rica Limon Costa Rica; ^3^ The Sloth Conservation Foundation Hayfield UK; ^4^ Present address: Department of Integrative Biology and Centre for Biodiversity Genomics University of Guelph 50 Stone Road E Guelph ON N1G 2W1 Canada; ^5^ Present address: Department of Integrative Biology University of Guelph 50 Stone Road E Guelph ON N1G 2W1 Canada

**Keywords:** agriculture, Costa Rica, ecology, gene pool, genotype, population genetics, reintroduction, sloths

## Abstract

Sloths are notoriously slow and consequently have limited dispersal ability, which makes them particularly vulnerable to the effects of habitat fragmentation and degradation. Sloths in Costa Rica are considered of conservation concern due to habitat loss, livestock production and increasing urbanization. Reintroductions from rescue centres are commonplace across the country, yet their genetic diversity and population structure are unknown, and there is currently little consideration of the genetic background prior to intervention or releases. We used microsatellite analysis to undertake the first exploratory investigation into sloth population genetics in Costa Rica. Using data from 98 two‐fingered sloths (*Choloepus hoffmanni*) from four different geographic regions, we determined the presence of four potential genetic groups, three of them with minimal population structuring despite the limited dispersal ability and presence of physical barriers. Sloths from the North appear to represent a highly distinct population that we propose may require management as a discrete unit for conservation. We stress the need for additional analyses to better understand the genetic structure and diversity of North andWest regions and suggest that rescue facilities in Costa Rica should consider the genetic background of rehabilitated sloths when planning future reintroductions. Our results also highlight the threat posed by physical isolation due to widespread urbanization and agriculture expansion for a species with a weak dispersal ability.

## 1 INTRODUCTION

Understanding the population structure of a species across recognized geographic scales (e.g. political, geological or ecological boundaries) is important for informing effective conservation management strategies, particularly for isolated populations that may represent distinct evolutionary lineages (Bickford et al., 2007; Coates, Byrne, & Moritz, 2018; Forcada & Hoffman, 2014; Moraes‐Barros, Miyaki, & Morgante, 2007; Ryder, 1986). Evolutionary significant units (ESUs) are classifications of populations based on their distinctness, which is often derived from a combination of ecological and genetic data, with a greater focus currently being placed on the molecular phylogenies of extant populations (Crandall, Bininda‐Emonds, Mace, & Wayne, 2000; Moritz, 1999; Vogler & Desalle, 1994). The extent to which phylogeographic differentiation occurs is largely dependent on the ecological and biological characteristics of each species, with mating systems, dispersal ability, habitat requirements and migration all significantly influencing gene flow (Bowman, Jaeger, & Fahrig, 2002; Edwards, Potter, Schmitt, Bragg, & Moritz, 2016; Whitmee & Orme, 2013). Natural barriers and anthropogenic landscape changes resulting in habitat fragmentation have been shown to influence genetic diversity of populations negatively (Olivieri, Sousa, Chikhi, & Radespiel, 2008; Schwartz, Luikart, & Waples, 2007). Genetic isolation of populations often causes higher rates of inbreeding and therefore can result in inbreeding depression, reducing the overall fitness of the population (Crnokrak & Roff, 1999; Frankham, 2010; Liberg et al., 2005). For small populations with low rates of dispersal, the effects of isolation can result in localized extinctions (Frankham, 2010; Fraser & Bernatchez, 2001; Palsbøll, Bérubé, & Allendorf, 2007).

Sloths are cryptic, arboreal mammals that spend much of their time resting high‐up in the dense rainforest canopy of South and Central America. Due to their low‐calorie folivorous diet, slow rate of digestion and low metabolic rate, sloths are critically limited by their rates of energy acquisition (Chiarello, 1998; Cliffe, Haupt, Avey‐Arroyo, & Wilson, 2015; Cliffe et al., 2018; Geiser, 2004; Irving, Scholander, & Grinnell, 1942; McNab, 1978; Nagy & Montgomery, 1980; Pauli, Peery, Fountain, & Karasov, 2016). They spend prolonged periods inactive, and when movement does occur, it is slow and deliberate to conserve energy and avoid predator detection (Goffart, 1971; Montgomery & Sunquist, 1975). As a consequence of their slow nature and highly specific arboreal lifestyle, sloths have a poor dispersal ability compared to other mammal species and are likely to be particularly sensitive to the fragmentation and disturbance of neotropical rainforests (Chiarello, 2008; Garcés‐Restrepo, Pauli, & Peery, 2018; Peery & Pauli, 2012, 2014). Annually, the deforestation rate for neotropical forests is 0.5%, with currently 55.8 million forest fragments existing across the Americas (Taubert et al., 2018). Sloths are physically unable to traverse gaps in the canopy by jumping, and moving on the ground is a laborious and dangerous strategy. As a result, even small levels of habitat fragmentation that cause a loss in canopy connectivity can hinder sloth dispersal and movement (Chiarello, 2008). In this context, the large‐scale fragmentation due to monoculture plantations, agriculture and widespread rainforest urbanization is of particular concern due to the complete isolation of sloth populations, which arises as a result (Sanchez‐Azofeifa, Harriss, & Skole, 2001).

In addition to habitat fragmentation through anthropogenic activities, natural forest structure and geography may act as barriers to dispersal for sloths across Costa Rica. There are 18 major river basins in Costa Rica: ten on the Atlantic slope and eight on the Pacific slope of the country (Sibaja, Bussing, Garita‐Alvarado, & López, 2013). However, considering that sloths are strong swimmers (Song, Chen, Chen, & Jia, 2016), it is unlikely that river systems are a barrier to dispersal for sloths in this case (van der Geer, Lyras, de Vos, & Dermitzakis, 2011). More likely, natural barriers to dispersal are the numerous mountain ranges, including the Central range, that extend from north‐west to south‐east of the country (Sánchez‐Murillo & Birkel, 2016). These ranges give rise to montane cloud forests observed throughout central Costa Rica (Kappelle, 2016). The genetic implications of sloth life history characteristics are poorly understood, and the influence of geographic barriers and habitat fragmentation on the genetic structure of wild sloth populations is currently unknown (Moraes‐Barros et al., 2007).

The adaptive differentiation of sloths across geographic regions is likely impacted by the sloth's ecological requirements in terms of temperature. Sloths have a reduced ability to maintain body temperature, presumably to help minimize thermogenic energy costs (Cliffe et al., 2018). The sloth's ecological niche is therefore thermally restricted to regions that maintain a relatively warm and stable temperature year‐round. While two‐fingered sloths (*Choloepus* sp.) can persist at moderately high altitudes, sloths in these regions have physiological and morphological adaptations to cope with a colder climate, including metabolic plasticity as well as longer, thicker and darker pelage compared to their lowland counterparts (Enders, 1940; McNab, 1985). Despite these adaptations, the colder temperatures at extreme elevations may represent a significant thermal barrier to dispersal (Zuloaga & Kerr, 2017), and the persistence and distribution of sloths in montane regions is poorly documented.

Sloths in Costa Rica are now considered of conservation concern due to habitat loss from agriculture (monocultures), livestock production and the increasing urbanization of the rainforest (including power line electrocutions, dog attacks and road traffic collisions; (Rodriguez‐Herrera, Chinchilla, & May‐Collado, 2002). Reflecting this, there has been a consistent increase in the number of sloths arriving at wildlife rescue facilities in recent years. There are two major wildlife rescue facilities in the South Caribbean region, which, between them, receive approximately 400 sloths per year. Considering their slow reproductive rate, the successful release of displaced sloths back into the wild is of growing importance for the conservation and management of the species. However, while sloth releases have become commonplace throughout Costa Rica, there is no existing legislation to encourage the consideration of an individual's genetic background prior to release and animals are not always returned to the location of origin. For example, in the past 20 years over 500 sloths have been returned to the wild in the South Caribbean. While many of these animals were rescued locally, some also originate from different regions around Costa Rica. It is currently unknown whether there are strong levels of population structuring across Costa Rica, and whether the relocation of sloths from different geographic locations into the same local area has any impact on the genetic diversity of the original or recipient populations.

To assess whether the low dispersal ability of sloths, combined with the presence of natural and anthropogenic barriers, has resulted in genetically divergent populations in different regions of Costa Rica, we have undertaken the first population analysis of sloth (*Choloepus* sp.) genetic structure in Costa Rica.

## 2 MATERIALS AND METHODS

### 2.1 Ethics statement

This research was approved by the Swansea University Animal Welfare & Ethical Review Process Group (AWERP), and the Costa Rican government and associated departments (MINAE, SINAC, ACLAC) permit numbers: R‐033‐2015 and R‐049‐2015. All research was performed in accordance with relevant guidelines and regulations.

### 2.2 Study sites and sample collection

All sampling was performed by a licensed veterinarian in the Sloth Sanctuary's veterinary clinic between September 2014 and November 2015. Between 30 and 50 hairs (including the follicular bulb) were collected from a total of 98 Hoffman's two‐fingered sloths (*Choloepus hoffmanni*) originating from numerous regions across Costa Rica (Table S1). All sloths sampled were born in the wild but were being maintained at the Sloth Sanctuary of Costa Rica for rehabilitation. The locations from which each sloth originated were obtained from sanctuary records. Hairs were collected from the lower back of each animal using tweezers, and all sloths were sampled under anaesthesia to minimize handling‐induced stress. Sampling took place during scheduled health checks, to avoid unnecessary sedation. 1 mg/kg of ketamine (Ketamina 50^®^, Holliday Scott) and 0.008 mg/kg of dexmedetomidine (Dexdomitor^®^, Zoetis) were administered intramuscularly by a licensed veterinarian following standardized sanctuary procedures. The anaesthesia was reversed using 0.008 mg/kg of anti‐sedative (atipamezole; Antisedan^®^, Zoetis). The hair samples were exported to Swansea University for analysis.

### 2.3 DNA extraction and amplification

DNA was extracted from between six and nine hair roots per sloth using 1‐Step DNA isolation kit (nexttec™) following the “Hair” protocol. The quality of extracted DNA was analysed using a Nanodrop 2000 (Thermo Fisher Scientific Inc.), and 10 μg was used for amplification. A total of 98 individuals were genotyped with 15 microsatellites (Table S3) in two multiplex reactions using the Qiagen Multiplex PCR kit, following the Qiagen multiplex reaction protocol (Qiagen). Each reaction consisted of 1.2 µl primer mix (0.6 µM each primer), 6 µl 2× QIAGEN Multiplex PCR Master Mix and 3 µl template DNA, made up to a total volume of 12 μl with ultrapure water.

Conditions for amplification consisted of a single‐cycle initial activation step of 15 min at 95°C followed by a touchdown PCR of eight cycles with a 30‐s denaturation step at 94°C, a 90‐s annealing step starting at 64°C and descending in 2 cycle steps of 2°C (64, 62, 60, 58 and 56°C) and 90‐s extension at 72°C. Following this, 24 additional cycles of PCR were run as above at an annealing temperature of 56°C followed by a single final extension cycle of 30 min at 60°C. Microsatellites were resolved on an Applied Biosystems ABI3130xl Genetic Analyser (Applied Biosystems), fragment length was determined using the GeneScan 500–LIZ size standard and scored using GeneMapper v45.0 (Applied Biosystems).

### 2.4 Genetic analyses

*Choloepus hoffmanni* individuals from 13 localities (Table S1) were pooled into four geographic regions (North, East, South‐East and West), as informed by Bayesian cluster analyses conducted in TESS (Chen, Durand, Forbes, & François, 2007) and phylogenetic analysis in POPTREEW (Takezaki, Nei, & Tamura, 2014), to ensure sufficient sample sizes for genetic analyses. Information regarding the land use and vegetation cover for these four geographic regions can be found in Table S2.

MICRO‐CHECKER v2.2.3 (Van Oosterhout, Hutchinson, Wills, & Shipley, 2004) was used to detect the presence of null alleles, large allele dropouts and scoring errors due to stuttering. GENALEX v6.5 (Peakall & Smouse, 2006, 2012) was used to estimate effective number of alleles (*N*_EF_) and the populations’ expected (*H*_E_) and observed heterozygosities (*H*_O_). In addition, GENALEX was also used to perform a Mantel test for isolation by distance (IBD), using standard parameters, including raw genetic and geographic values, with geographic distance measured in kilometres. Conformity to Hardy–Weinberg expectations of genotype frequencies and tests for linkage disequilibrium was investigated using GENEPOP online v4.0.10 (Raymond & Rousset, 1995; Rousset, 2008). Exact tests of differences in allele frequencies and significance of pairwise *F_ST_* values were calculated using FSTAT v1.2 (Goudet, 1995). Analysis of molecular variance (AMOVA) among populations, among individuals and within individuals was calculated in ARLEQUIN v3.5.2.2 (Excoffier & Lischer, 2010). Homozygosity by locus (HL) was estimated for each individual in Cernicalin v1.0 (Aparicio, Ortego, & Cordero, 2006).

Prior to assessing patterns of population structure, a phylogenetic tree was constructed based on the uncorrected *F_ST_* values for *C. hoffmanni* at each locality (Aviarios, Bribri, Cahuita, Guacimo, Guapiles, Limón, Limoncito, Penshurt, Puerto Viejo, Río Banano, San José, Siquirres and Turrialba) using the neighbour‐joining method with 999 bootstrap iterations in POPTREEW.

To assess how best to pool the individuals for further analyses, we conducted Bayesian cluster analysis in TESS v2.3.1. All sloths were assigned to a site during sample collection; however, some individuals’ locations were also recorded using GPS coordinates. To generate the individual coordinates required for analysis, TESS randomly assigned each sloth coordinates within geographic ranges informed by the minimum and maximum latitudes and longitudes of GPS coordinates recorded at each respective site. Admixture models were run with 50,000 total sweeps, 10,000 burn‐in sweeps and 200 runs per *K*_max_ from *K* = 2 to 13, following standard suggested parameters. The most likely number of clusters was assessed using the average deviance information criterion (DIC) of the lowest 10 DIC values for each *K*_max_. Results of most likely K were visualized using STRUCTURE (Pritchard, Stephens, & Donnelly, 2000) and Structure Selector (Li & Liu, 2018).

Patterns of gene flow between pooled groups were assessed in divMigrate software (Sundqvist, Keenan, Zackrisson, Prodöhl, & Kleinhans, 2016), using the *N*_m_ statistic described by Alcala, Goudet, & Vuilleumier (2014) to calculate the number of effective migrants. The directional asymmetry of gene flow between populations was calculated using 5,000 bootstrap simulations.

BARRIER v2.2 (Manni, Guérard, & Heyer, 2004) was used to detect barriers to gene flow resulting in discontinuities in allelic frequencies between sloth populations, based on genetic distance and geographic distance values using the Monmonier's maximum difference algorithm (Monmonier, 1973). Initially, one data matrix containing pairwise *F*_ST_ values for the pooled groups was imported in BARRIER to detect genetic barriers across all populations. Fifteen data matrices were then imported into BARRIER containing pairwise *F*_ST_ values per locus to assess the number of loci supporting each barrier and test for barrier robustness (Manni et al., 2004).

## 3 RESULTS

### 3.1 Genetic diversity and gene flow

MICRO‐CHECKER yielded no evidence of null alleles across any loci for all populations. Tests for linkage disequilibrium using Fisher's method for each locus pair across all populations did not show any significant associations over 105 pairwise comparisons after sequential Bonferroni corrections for multiple tests. All 15 microsatellite loci displayed a high degree of polymorphism (average *H*_E_ = 0.67). There was evidence of significant deviation from Hardy–Weinberg equilibrium (HWE) across various loci, though out of 60 chi‐squared tests only 11.6% of loci deviated significantly from HWE after Bonferroni corrections (Table S3). At a population level, there was no evidence of significant deviations from HWE (Table S3).

The allelic richness (*N*_a_) varied over the four populations, with the South‐East (5.67) having the highest and the West (4.53) the lowest (Table 1). Mean expected heterozygosity (*H*_E_) ranged from 0.62 to 0.69. The West population had the lowest mean number of effective alleles (*N*_EF_) of 2.95, whereas the South‐East had the highest *N*_EF_ of 3.39. Results of a one‐way ANOVA revealed no significant difference in *H*_e_ (*F*_3,56_ = 0.992, *p* = .403) nor *N*_EF_ (*F*_3,56_ = 1.797, *p* = .158) between populations.

**TABLE 1:** Summary statistics for each geographic population of *Choloepus hoffmanni*

	East	South‐East	West	North
*N*	25	43	10	20
*N*_a_ (±*SE*)	5.27 (0.43)	5.67 (0.33)	4.60 (0.32)	4.53 (0.39)
*N*_EF_ (±*SE*)	3.36 (0.26)	3.39 (0.21)	2.93 (0.24)	2.95 (0.30)
*N*_PA_ (±*SE*)	0.33 (0.16)	0.53 (0.19)	0.07 (0.07)	0.27 (0.12)
*H*_O_ (±*SE*)	0.54 (0.04)	0.62 (0.03)	0.44 (0.04)	0.59 (0.04)
*H*_E_ (±*SE*)	0.68 (0.02)	0.69 (0.03)	0.63 (0.03)	0.62 (0.03)
*H*_L_ (±*SE*)	0.46 (0.03)	0.38 (0.02)	0.56 (0.06)	0.41 (0.04)
*F*_IS_ (±*SE*)	0.20 (0.05)	0.34 (0.10)	0.27 (0.06)	0.04 (0.05)
*HW* (±*SE*)	0.20 (0.07)	0.09 (0.04)	0.25 (0.06)	0.52 (0.08)

Abbreviations: *F*_IS_, mean fixation index; *H*_E_, mean expected heterozygosity; *H*_L_, mean homozygosity by locus; *H*_O_, mean observed heterozygosity; *N*, number of individuals; *N*_A_, mean number of alleles; *N*_EF_, mean number of effective alleles; NPA, mean number of private alleles.

There was considerable variation in effective number of migrants (*N*_m_) calculated between sites, ranging from 1.00 between the two Eastern groups and 0.161 between the South‐East and Northern groups (Table 2). Though there was high migrant exchange between the East and South‐East, only the East showed moderate gene flow across to San José in the West. The Northern group appeared relatively isolated from the East, though it too showed moderate migrant exchange with the West. There was no evidence of asymmetric gene flow across any of the groups (Table 3).

**TABLE 2:** Effective number of migrants (*Nm*) between four genetic groups of *Choloepus hoffmanni* sampled in Costa Rica estimated, using divMigrate across 15 microsatellite loci

	East	South‐East	West	North
East	0.000	0.889	0.693	0.211
South‐East	1.000	0.000	0.478	0.161
West	0.748	0.335	0.000	0.353
North	0.326	0.280	0.539	0.000

**TABLE 3:** Pairwise *F*_ST_ values (below diagonal) and significance (above diagonal) for four groups of *Choloepus hoffmanni* sampled in Costa Rica

	East	South‐East	West	North
East	0.00	*	—	*
South‐East	0.03	0.00	*	*
West	0.02	0.07	0.00	*
North	0.09	0.14	0.04	0.00

Note

Significance values for each pairwise comparison adjusted by sequential Bonferroni's corrections *p *<* *.00045.

### 3.2 Population structuring and homozygosity by locus

Global *F*_ST_ (0.06) and pairwise *F*_ST_ values were significant (after adjusting for Bonferroni's corrections, *p* < .00045), albeit very low in general, except for the pairwise comparisons between East and West (Table 3). The highest divergence was observed between the North and South‐East populations (*F*_ST_ = 0.14), with the lowest divergence between West and East (*F*_ST_ = 0.02). Most populations displayed a high degree of admixture, with average *Q* values ranging between 2% and 20%, apart from the South‐East that had an average *Q* value of 73%. The AMOVA results for all populations suggested that the greatest degree of genetic differentiation was within individuals (75%), with differentiation among populations and among individuals accounting for 5% and 22%, respectively (Table 4). Results of the Mantel test (using central latitude and longitude per site) did not support isolation by distance (IBD), despite some sites being geographically distant (i.e. East and West; *y* = 0.0104*x* + 26.889, *R* ^2^ = 0.0148, *p* > .05).

**TABLE 4:** Results of analysis of molecular variance (AMOVA) for the four groups of *Choloepus hoffmanni*, presenting the different sources of variation (among populations, among individuals, within individuals), degrees of freedom (*df*), sum of squared differences (SSD), variance components, percentage variation and *p* value for each source

Source	*df*	SSD	Variance components	Percentage variation	*F*‐statistics	*p* value
Among populations	3	56.31	0.27	5.00	0.048	<.001
Among individuals	94	619.81	1.25	22.00	0.235	<.001
Within individuals	98	400.50	4.09	73.00	0.272	<.001

**FIGURE 1:**
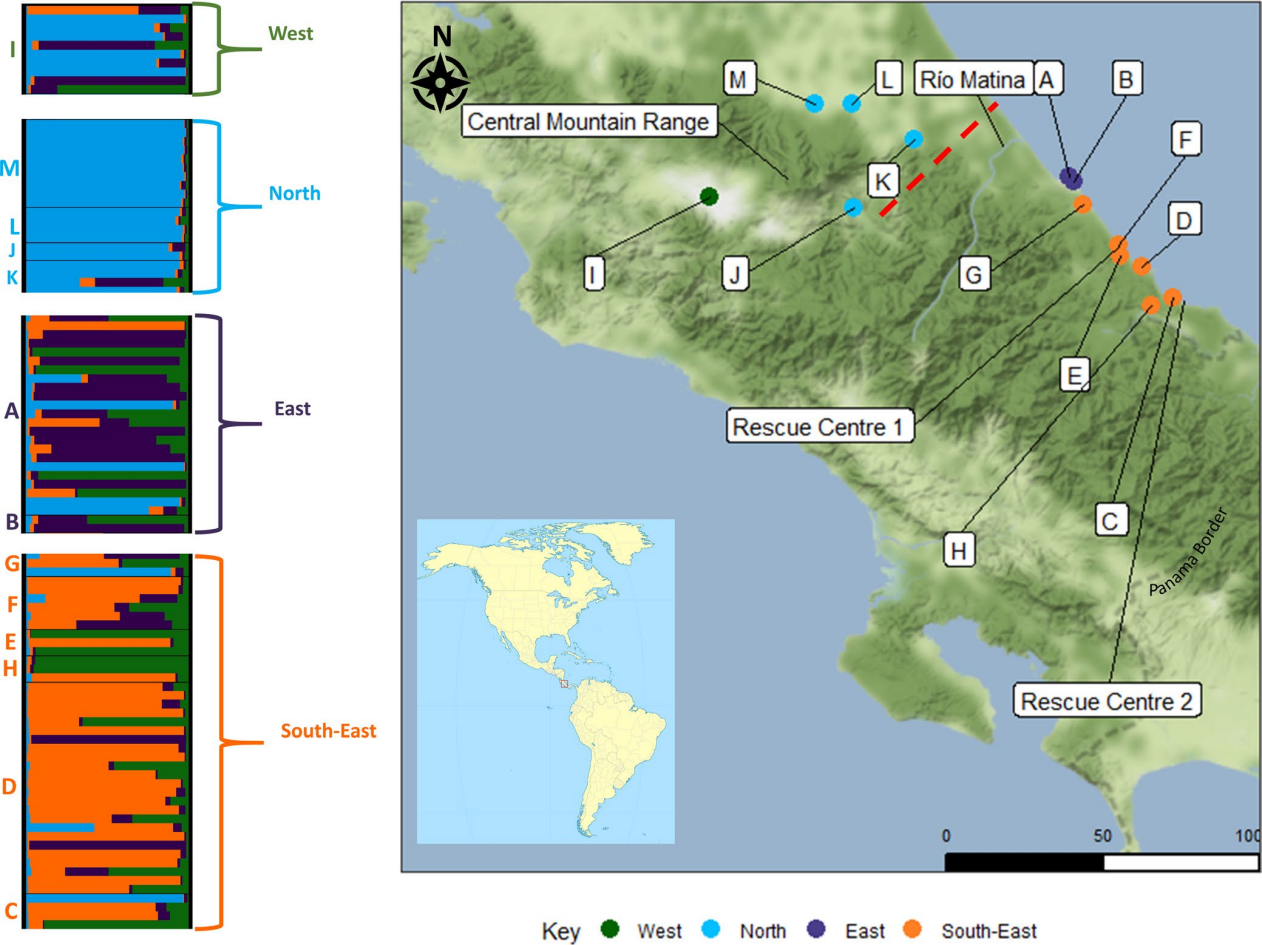
Terrain map of sample sites in Costa Rica where 98 individual *Choloepus hoffmanni* sloths were hair sampled for genetic analyses. Scale bar is measured in kilometres. Groupings of sloths and the locations of rescue centres, the Matina River and the Central mountain range are indicated in the key. Output from Structure Selector (*K* = 4) is displayed to the left, with site locations labelled. The main significant break in genetic discontinuity (calculated in BARRIER) is indicated by a red dotted line (break supported by 8 out of 15 loci). A, Limón; B, Limoncito; C, Puerto Viejo; D, Cahuita; E, Penshurt, F, Aviarios; G, Río Banano; H, Bribri; I, San José; J, Turrialba; K, Siquirres; L, Guacimo; M, Guapiles

**FIGURE 2:**
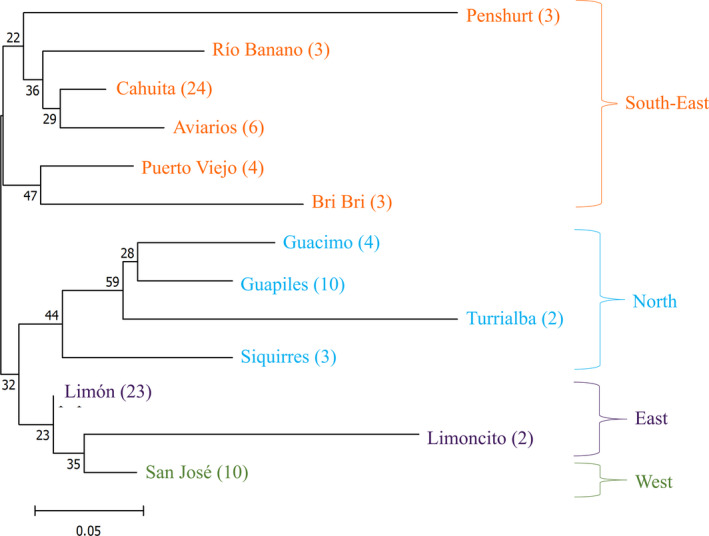
Phylogenetic tree for 98 individual *Choloepus hoffmanni* from 13 localities. Tree constructed in POPTREE 3 using neighbour‐joining method with 999 Bootstrap iterations. Number next to locality indicates number of individuals, with colours referring to geographic grouping. The number at each node refers to Bootstrap value, as the proportion of total replications

The analysis conducted in TESS suggested a *K*_max_ of 8; however, four of the groups only contributed 6.97% to the genetic background. The most likely number of genetic clusters (*K*) was 4 (Figure 1; Figures S1 and S2). Common genetic profiles were identified across sites in the West (San José), North (Guacimo, Guapiles, Siquirres, Turrialba), East (Limón, Limoncito) and South‐East (Aviarios, Bribri, Cahuita, Penshurt, Puerto Viejo, Río Banano). The phylogenetic tree revealed two main clades: 1: South‐East and 2: North, East and West, with East and West being in the same subclade (Figure 2). Despite bootstrap values being lower than 50 for most nodes, the co‐ancestry presented is in line with our geographic groupings, as inferred through TESS.

Results from BARRIER suggested that the least number of barriers present to capture the breaks in genetic discontinuities between the four populations was one barrier (Figure 1; Figure S3). This division occurred between North and East (+South‐East) populations, supported by eight loci.

Homozygosity by locus (*H*_L_) varied across the four groups, with the West having the highest degree of homozygosity (0.56) and the South‐East the lowest (0.38; Table 1). Within the South‐East group, two individuals genotyped were reported to have physical deformities (i.e. missing/shortened limbs and digits), and their individual *H*_L_ values were 0.55 and 0.49. Across the populations, results of a one‐way ANOVA revealed a significant difference of *H*_L_ (*F*_3,94_ = 3.894, *p* < .05), between the West and South‐East populations (Tukey HSD; adjusted *p*‐value = 0.01).

## 4 DISCUSSION

In this study, we investigated the genetic diversity and population structure of *C. hoffmanni* sloths in regions of Costa Rica and revealed relatively high genetic diversity and almost nonexistent population structuring apart from separation seen with the North grouping. Despite the presence of natural barriers and the known limited dispersal of sloths, the lack of isolation by distance and low levels of genetic differentiation between groupings was unexpected.

The home range size and natural dispersal habits of sloths vary widely between individuals, with some sloths being known to disperse over four hectares (Vaughan, Ramírez, Herrera, & Guries, 2007), while other individuals rarely move from a single tree (Pauli, Peery, Hayden, Pemberton, & Herrera, 2012; Peery & Pauli, 2012). This variance makes defining the ranges of natural sloth populations in a neotropical rainforest challenging. We therefore used Bayesian clustering analysis to determine the most likely number of genetic groups of *C. hoffmanni* represented in samples collected (Chen et al., 2007).

Despite the overall uniformity of sloth groups across Costa Rica, the Northern group was the most genetically divergent in comparison to the remaining three groups. The Matina River (Río Matina), which flows into the Caribbean on the eastern coast (Kohlmann, 2012), is located between the Northern population and the East and South‐East groups. Based on prior knowledge of the swimming ability of sloths (Song et al., 2016) and the relatively low *F*_ST_ value, this river is unlikely to be the cause of the barrier to geneflow observed (Slatkin, 1987). Based on our results, and considering the physiological and morphological adaptations of sloths to different climatic regions (Enders, 1940; McNab, 1985), it may be necessary to recognize sloths within the North group as a discrete conservation unit for management and conservation purposes. However, more in‐depth work using the data presented in this study should be undertaken to test the altitudinal adaptation theory with geospatial and morphological data.

Despite the presence of the Central mountain range, there was evidence of gene flow between the East and West groups, combined with a lack of significant population structuring in the West population (Tiffin & Ross‐Ibarra, 2014). However, within the individual sloths in the West group there was high average homozygosity by locus (*H*_L_ = 0.56), indicating higher levels of inbreeding (*F*_IS_ = 0.27), which is expected as sloths in this area are limited to severely fragmented forest pockets within the highly urbanized San José region.

Compared to the other three groups, the South‐East grouping (Aviarios, Bribri, Cahuita, Penshurt, Puerto Viejo and Río Banano) was more genetically diverse, with high levels of admixture and lack of population structuring. Located in the South‐East are wildlife rescue facilities, that release rehabilitated sloths, originating from across Costa Rica, into the local area. The reintroduction of many individuals (likely with different genetic origins) into the local area may have contributed to the nonexistent population structuring and higher levels of genetic diversity observed (Banes, Galdikas, & Vigilant, 2016; Johnson, 2000). The long‐term effects of translocations of individuals from their area of origin into the South‐East region are unknown.

Continuing to release rehabilitated northern and western individuals in the South‐East region (either due to a lack of suitable habitat in natal range or through a lack of proper support and funding for rescue centres) may result in lack of sufficient genetic diversity to maintain viability of populations located in the North (Lacy, 1997; Weeks et al., 2011). Removing individuals to release elsewhere could result in a gradual bottleneck effect in the North (Lacy, 1997). The northern population is however more vulnerable than the West due to the higher level of unique genetic diversity (i.e. number of private alleles). In addition, there is the risk that unique local adaptations will be lost when relocating sloths (Weeks et al., 2011). Similar concerns have recently been raised for orangutans in Tanjung Puting National Park (Borneo), which have been reintroduced to the wild from rescue facilities without knowledge on the genetic background and subspecies status of the individuals (Banes et al., 2016). In Costa Rica, this is also of particular concern given the unique genetic structure in the North group and the physiological/morphological adaptations shown by sloths originating from cooler montane regions (Enders, 1940; McNab, 1985).

It was not possible to determine population genetic structure prior to sloth reintroduction in Costa Rica. Therefore, it is difficult to determine whether the current genetic uniformity and lack of isolation by distance observed are largely natural or due to translocations of individuals; however, this study will act as a baseline for future research. Additionally, due to sample size limitations, our South‐East group spans sloths from many different habitat fragments within the region. It is possible that by combining these subclusters together as a single population, we could be masking the true levels of diversity in current analyses. This effect may not be present in other regions, or perhaps was not identified due to lower sample sizes. While our data do not allow to draw firm conclusions on the driving factors behind the high level of admixture observed across Costa Rica, future work could include the use of SNPs or other fine‐scale genomic analyses (Morin, Luikart, & Wayne, 2004; Ouborg, Pertoldi, Loeschcke, Bijlsma, & Hedrick, 2010), to further investigate gene flow within and between “relocated” versus “relocated and native” populations. The analyses presented in this study form baseline information on existing sloth genotypes and provide evidence that sloths are not being returned to their point of origin, which is important for implementing policy regarding sloth reintroductions. However, further in‐depth analyses are required to determine the extent of barriers and drivers on the genetic structure of sloths in the studied regions of Costa Rica.

Anthropogenic pressures on sloth populations in Costa Rica are increasing (Neam & Lacher, 2018; Rodriguez‐Herrera et al., 2002). Current populations are at risk of further fragmentation and isolation through the expansion of monocultures and urban sprawl, which will ultimately see an increase in the number of sloths needing to be rescued, rehabilitated and reintroduced. There is an emerging global awareness of the need to consider the genotypes of reintroduction candidates, recipient populations and origin populations prior to release, including official guidelines and recommendations set out by the International Union for the Conservation of Nature (IUCN/SSC, 2013). However, in Costa Rica, there are no existing protocols or legislation to encourage this practice. Our results support the proposal to consider sloths within the North group as a discrete subpopulation (or ESU), and further studies should therefore be focussed in the North and West regions to investigate in detail the existing genetic diversity of highly structured populations and to determine the fitness of populations isolated through urbanization, to better understand the long‐term effects of habitat fragmentation on *C. hoffmanni* in Costa Rica.

## AUTHORS CONTRIBUTIONS

R.N.C., R.P.W., J.A.A. and S.C. conceived and designed the study. R.N.C., S.J.K. and J.A.A. collected the samples. R.N.C., C.V.R. and B.A.W. carried out the genetic analysis. C.V.R. and B.A.W. carried out the statistical analysis. All contributed to writing and reviewing the manuscript. Authors declare no conflict of interest.

## Supporting information

Supplementary Material

## Data Availability

Full genotypes for all 98 individuals can be found in supporting information file.
