# Supplementary material for: Genetic divergence and evidence of human‐mediated translocation of two‐fingered sloths (Choloepus hoffmanni) in Costa Rica
Source: Evol Appl. 2020 Jun 26;13(9):2439–48. doi: 10.1111/eva.13036 (PMC7513709; doi:10.1111/eva.13036)
Supplement: Supplementary file 1 — Supplementary Material [file EVA-13-2439-s001.docx]

**Table S1**. Information of 13 localities at which *Choloepus hoffmanni* hair samples were obtained, including the number of individuals sampled per site (*N*), minimum and maximum longitude and latitude recorded at each site, and the genetic region as identified by structure analysis in TESS.

| **Site** | **Region** | ***N*** | **Min Long.** | **Max Long.** | **Min Lat.** | **Max Lat.** |
| --- | --- | --- | --- | --- | --- | --- |
| Limon | East | 23 | 8.827 | 10.459 | -84.823 | -82.393 |
| Limoncito | East | 2 | 8.827 | 8.828 | -83.017 | -83.016 |
| Puerto Viejo | South East | 4 | 9.392 | 9.393 | -82.467 | -82.466 |
| Cahuita | South East | 24 | 9.443 | 9.446 | -83.435 | -82.506 |
| Penshurt | South East | 3 | 9.463 | 9.466 | -82.546 | -82.543 |
| Aviarios | South East | 6 | 9.471 | 9.476 | -82.550 | -82.545 |
| Rio Banano | South East | 3 | 9.551 | 9.554 | -82.230 | -83.111 |
| Bri Bri | South East | 3 | 9.628 | 9.629 | -82.817 | -82.816 |
| San Jose | West | 10 | 9.551 | 10.290 | -84.559 | -84.112 |
| Turrialba | North | 2 | 9.582 | 9.583 | -83.385 | -83.384 |
| Siquirres | North | 4 | 10.102 | 10.103 | -83.507 | -83.506 |
| Guacimo | North | 4 | 10.124 | 10.125 | -83.419 | -83.411 |
| Guapiles | North | 10 | 10.171 | 10.175 | -83.464 | -83.435 |

**Table S2.** Summary table of the primary, secondary and other land use/vegetation cover types within the four groupings of *Choloepus hoffmanni* individuals.

| **Grouping** | **Primary land use/vegetation cover** | **Secondary land use/vegetation cover** | **Other land use/ vegetation cover types** | **Reference** |
| --- | --- | --- | --- | --- |
| North | Tropical wet forest | Native regrowth | Pineapple plantation and pasture | (Fagan et al., 2013; Pontius, Cornell, & Hall, 2001) |
| East | Tropical premontane forest | Pasture | N/A | (Carlson & Sanchez-Azofeifa, 1999; Pontius et al., 2001) |
| South-East | Premontane wet forest | Tropical moist forest | Pasture | (Jadin, Meyfroidt, & Lambin, 2016; Pontius et al., 2001) |
| West | Urban | Pasture | Tropical moist forest | (Carlson & Sanchez-Azofeifa, 1999; Öborn et al., 2013; Pontius et al., 2001; Van Laake & Sánchez-Azofeifa, 2004) |

**Table S3.** Genetic diversity values of four groupings of *Choloepus hoffmanni* for 15 microsatellite loci. *N*_A_ Number of alleles; *N*_E_ Number of effective alleles; *H*_O_ Observed heterozygosity; *H*_E_ Expected heterozygosity; *H*_W_ *P*-values for deviation of Hardy-Weinberg equilibrium; *F_IS_* Fixation index (positive value indicates homozygosity excess); N Number of samples. Significant values of deviation of HW after Bonferroni correction (*P* <0.0000617) are indicated in bold.

|  |  | **Microsatellite** | | | | | | | | | | | | | | |
| --- | --- | --- | --- | --- | --- | --- | --- | --- | --- | --- | --- | --- | --- | --- | --- | --- |
| **Population** |  | **A1** | **B1** | **B2** | **C1** | **C2** | **D1** | **D2** | **Aa** | **Ab** | **Ba** | **Bb** | **Ca** | **Cb** | **Da** | **Db** |
| **East** | ***N*_A_** | 4 | 4 | 5 | 4 | 5 | 4 | 9 | 4 | 8 | 5 | 5 | 5 | 8 | 5 | 4 |
|  | ***N*_E_** | 2.665 | 3.446 | 3.418 | 2.934 | 2.665 | 2.873 | 5.675 | 2.141 | 4.630 | 3.470 | 3.213 | 2.660 | 5.020 | 3.524 | 2.129 |
|  | ***H*_O_** | 0.571 | 0.609 | 0.208 | 0.520 | 0.560 | 0.476 | 0.625 | 0.500 | 0.760 | 0.625 | 0.400 | 0.400 | 0.720 | 0.750 | 0.400 |
|  | ***H*_E_** | 0.625 | 0.710 | 0.707 | 0.659 | 0.625 | 0.652 | 0.824 | 0.533 | 0.784 | 0.712 | 0.689 | 0.624 | 0.801 | 0.716 | 0.530 |
|  | ***H*_W_** | 0.453 | 0.009 | **0.000** | 0.412 | 0.906 | 0.071 | **0.000** | 0.461 | 0.354 | 0.137 | 0.011 | 0.130 | **0.000** | 0.031 | **0.000** |
|  | ***F*_IS_** | 0.085 | 0.142 | 0.706 | 0.211 | 0.104 | 0.270 | 0.241 | 0.062 | 0.031 | 0.122 | 0.419 | 0.359 | 0.101 | -0.047 | 0.246 |
| **South East** | ***N*_A_** | 3 | 4 | 5 | 6 | 5 | 5 | 7 | 6 | 7 | 6 | 5 | 7 | 8 | 6 | 5 |
|  | ***N*_E_** | 2.278 | 2.648 | 3.059 | 2.806 | 2.878 | 3.979 | 4.290 | 3.239 | 5.080 | 4.096 | 3.196 | 3.824 | 4.136 | 3.196 | 2.159 |
|  | ***H*_O_** | 0.622 | 0.528 | 0.450 | 0.442 | 0.605 | 0.676 | 0.825 | 0.575 | 0.558 | 0.789 | 0.581 | 0.837 | 0.674 | 0.707 | 0.488 |
|  | ***H*_E_** | 0.561 | 0.622 | 0.673 | 0.644 | 0.653 | 0.749 | 0.767 | 0.691 | 0.803 | 0.756 | 0.687 | 0.739 | 0.758 | 0.687 | 0.537 |
|  | ***H*_W_** | 0.745 | **0.000** | 0.037 | **0.000** | 0.011 | 0.791 | 0.648 | 0.342 | 0.012 | 0.937 | 0.023 | 0.249 | 0.360 | 0.908 | **0.000** |
|  | ***F*_IS_** | -0.108 | 0.152 | 0.331 | 0.313 | 0.073 | 0.096 | -0.076 | 0.168 | 0.305 | -0.044 | 0.154 | -0.134 | 0.111 | -0.029 | 0.090 |
| **West** | ***N*_A_** | 3 | 4 | 5 | 4 | 3 | 4 | 6 | 3 | 6 | 4 | 5 | 6 | 7 | 4 | 5 |
|  | ***N*_E_** | 1.684 | 2.656 | 4.378 | 2.597 | 2.597 | 2.945 | 5.226 | 2.174 | 2.222 | 2.513 | 2.893 | 3.279 | 3.774 | 2.282 | 2.703 |
|  | ***H*_O_** | 0.375 | 0.333 | 0.556 | 0.400 | 0.300 | 0.444 | 0.778 | 0.200 | 0.400 | 0.714 | 0.222 | 0.400 | 0.500 | 0.444 | 0.600 |
|  | ***H*_E_** | 0.406 | 0.623 | 0.772 | 0.615 | 0.615 | 0.660 | 0.809 | 0.540 | 0.550 | 0.602 | 0.654 | 0.695 | 0.735 | 0.562 | 0.630 |
|  | ***H*_W_** | 0.409 | 0.268 | 0.740 | 0.081 | 0.095 | 0.225 | 0.340 | 0.006 | 0.181 | 0.247 | 0.048 | 0.054 | 0.037 | 0.402 | 0.609 |
|  | ***F*_IS_** | 0.077 | 0.465 | 0.280 | 0.350 | 0.512 | 0.327 | 0.038 | 0.630 | 0.273 | -0.186 | 0.660 | 0.424 | 0.320 | 0.209 | 0.048 |
| **North** | ***N*_A_** | 2 | 3 | 6 | 6 | 3 | 4 | 6 | 3 | 7 | 6 | 5 | 4 | 5 | 5 | 3 |
|  | ***N*_E_** | 1.519 | 2.623 | 3.252 | 3.292 | 1.946 | 1.847 | 4.624 | 1.982 | 5.926 | 3.406 | 2.649 | 2.186 | 3.433 | 2.906 | 2.694 |
|  | ***H*_O_** | 0.438 | 0.500 | 0.400 | 0.800 | 0.450 | 0.316 | 0.750 | 0.500 | 0.750 | 0.789 | 0.650 | 0.600 | 0.700 | 0.500 | 0.650 |
|  | ***H*_E_** | 0.342 | 0.619 | 0.693 | 0.696 | 0.486 | 0.458 | 0.784 | 0.495 | 0.831 | 0.706 | 0.623 | 0.543 | 0.709 | 0.656 | 0.629 |
|  | ***H*_W_** | 0.263 | 0.261 | 0.226 | 0.649 | 0.854 | 0.026 | 0.036 | 0.765 | 0.758 | 0.800 | 0.569 | 0.973 | 0.601 | 0.557 | 0.454 |
|  | ***F*_IS_** | -0.280 | 0.192 | 0.422 | -0.149 | 0.075 | 0.311 | 0.043 | -0.009 | 0.098 | -0.118 | -0.044 | -0.106 | 0.012 | 0.238 | -0.034 |


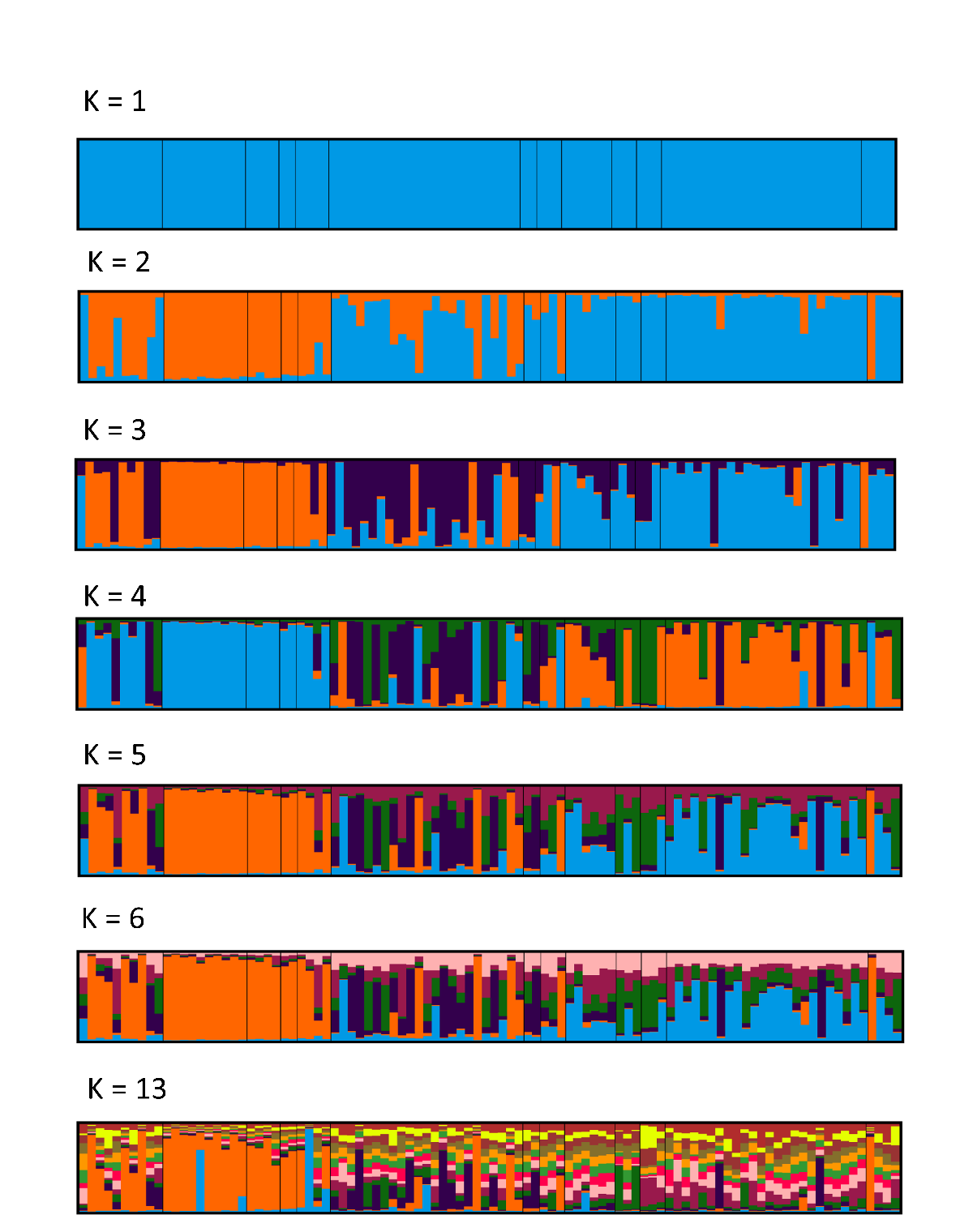


**Figure S1.** Output of different K plots from STRUCTURE SELECTOR.


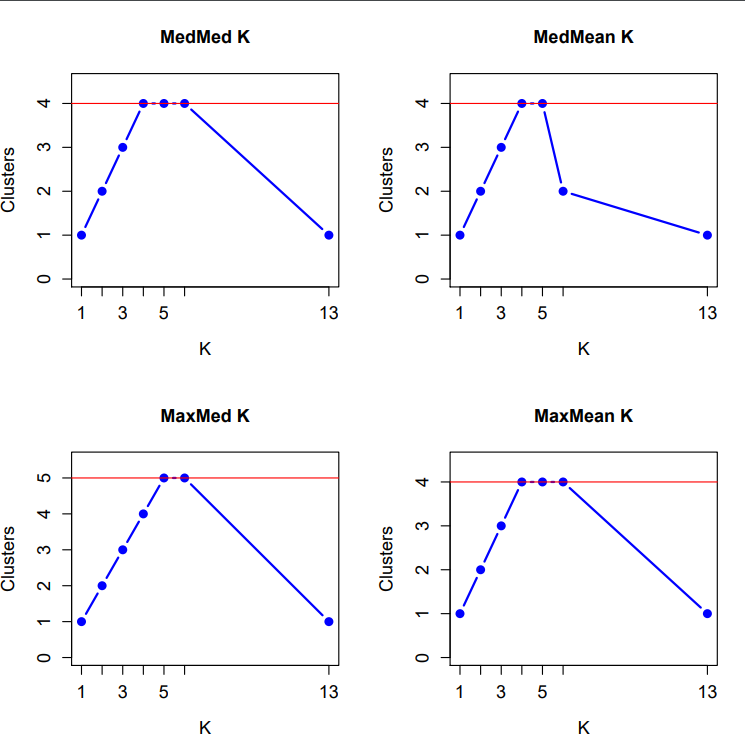


**Figure S2.** The optimal K (Y-axis) after removing spurious clusters, indicated by red lines (i.e. the number of clusters) used to explain predefined 13 populations. Threshold=0.5.


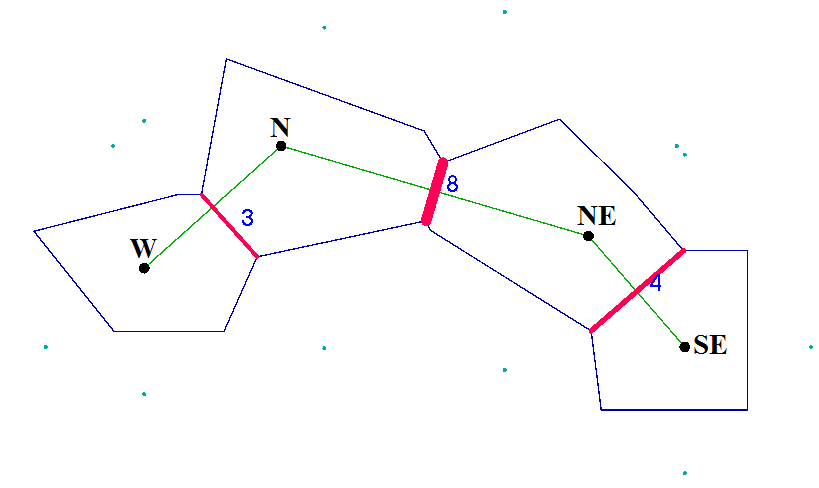


**Figure S3.** Output from BARRIER, highlighting the main break in genetic continuity of four groupings of 98 *Choloepus hoffmanni* individuals (red lines); the green line corresponds to the Delaunay triangulation between populations (red dots) and Voronoï tessellation used to calculate position of the barriers (blue line). Populations: E = East; SE = South East; W = West; N = North. The most likely number of barriers identified was one (break between North and East), supported by 8 loci.

Appendix A

|  | | | **Microsatellite** | | | | | | | | | | | | | | | | | | | | | | | | | | | | | |
| --- | --- | --- | --- | --- | --- | --- | --- | --- | --- | --- | --- | --- | --- | --- | --- | --- | --- | --- | --- | --- | --- | --- | --- | --- | --- | --- | --- | --- | --- | --- | --- | --- |
| **Sloth_ID** | **Population** | **Location** | **A1** | | **B1** | | **B2** | | **C1** | | **C2** | | **D1** | | **D2** | | **Aa** | | **Ab** | | **Ba** | | **Bb** | | **Ca** | | **Cb** | | **Da** | | **Db** | |
| 1 | East | Limón | 150 | 150 | 237 | 237 | 292 | 292 | 184 | 188 | 262 | 274 | 197 | 197 | 274 | 278 | 145 | 145 | 272 | 268 | 276 | 284 | 228 | 236 | 294 | 294 | 224 | 236 | 298 | 306 | 258 | 266 |
| 2 | East | Limón | 158 | 158 | 234 | 237 | 292 | 296 | 184 | 184 | 258 | 274 | 201 | 201 | 274 | 286 | 133 | 145 | 276 | 280 | 280 | 288 | 240 | 240 | 286 | 294 | 236 | 236 | 306 | 306 | 258 | 258 |
| 3 | East | Limón | 150 | 150 | 234 | 237 | 292 | 292 | 188 | 188 | 258 | 258 | 185 | 201 | 274 | 274 | 141 | 141 | 268 | 284 | 280 | 280 | 228 | 228 | 290 | 290 | 236 | 236 | 298 | 302 | 258 | 266 |
| 4 | East | Limón | 150 | 158 | 234 | 237 | 288 | 288 | 184 | 188 | 258 | 258 | 197 | 197 | 274 | 290 | 141 | 145 | 268 | 268 | 284 | 284 | 236 | 236 | 290 | 298 | 220 | 220 | 298 | 306 | 258 | 258 |
| 5 | East | Limón | 150 | 150 | 0 | 0 | 304 | 304 | 204 | 204 | 258 | 258 | 197 | 197 | 270 | 282 | 141 | 141 | 268 | 276 | 276 | 276 | 236 | 244 | 274 | 294 | 232 | 240 | 298 | 302 | 254 | 258 |
| 6 | East | Limón | 150 | 154 | 234 | 237 | 288 | 288 | 188 | 188 | 258 | 274 | 197 | 197 | 282 | 286 | 137 | 141 | 268 | 276 | 280 | 284 | 228 | 232 | 290 | 290 | 228 | 236 | 294 | 302 | 258 | 258 |
| 7 | East | Limón | 154 | 158 | 228 | 228 | 288 | 296 | 188 | 204 | 274 | 274 | 185 | 201 | 278 | 282 | 133 | 145 | 264 | 280 | 0 | 0 | 236 | 240 | 274 | 274 | 232 | 240 | 298 | 310 | 258 | 258 |
| 8 | East | Limón | 150 | 150 | 231 | 234 | 292 | 292 | 188 | 204 | 258 | 274 | 189 | 197 | 278 | 286 | 141 | 141 | 268 | 268 | 280 | 284 | 228 | 228 | 290 | 294 | 224 | 240 | 294 | 302 | 258 | 258 |
| 9 | East | Limón | 158 | 162 | 234 | 237 | 288 | 288 | 188 | 196 | 258 | 270 | 197 | 197 | 170 | 174 | 141 | 145 | 280 | 284 | 280 | 280 | 232 | 244 | 274 | 290 | 220 | 240 | 0 | 0 | 254 | 258 |
| 10 | East | Limón | 150 | 158 | 231 | 234 | 288 | 288 | 188 | 188 | 258 | 270 | 0 | 0 | 270 | 282 | 141 | 145 | 268 | 280 | 280 | 284 | 228 | 240 | 290 | 290 | 220 | 224 | 0 | 0 | 258 | 266 |
| 11 | East | Limón | 150 | 158 | 228 | 231 | 292 | 296 | 188 | 196 | 274 | 274 | 189 | 197 | 278 | 278 | 141 | 141 | 264 | 284 | 280 | 292 | 236 | 240 | 290 | 290 | 232 | 236 | 294 | 302 | 258 | 258 |
| 12 | East | Limón | 150 | 158 | 237 | 237 | 288 | 296 | 188 | 204 | 258 | 270 | 197 | 201 | 270 | 286 | 141 | 141 | 264 | 280 | 284 | 288 | 228 | 228 | 274 | 274 | 236 | 240 | 302 | 310 | 258 | 266 |
| 13 | East | Limón | 0 | 0 | 0 | 0 | 292 | 292 | 184 | 184 | 258 | 274 | 0 | 0 | 270 | 282 | 137 | 141 | 260 | 272 | 280 | 288 | 240 | 240 | 290 | 290 | 236 | 236 | 298 | 298 | 266 | 266 |
| 14 | East | Limón | 158 | 158 | 234 | 237 | 292 | 292 | 184 | 188 | 258 | 258 | 197 | 197 | 274 | 290 | 141 | 141 | 268 | 276 | 284 | 284 | 228 | 228 | 294 | 298 | 220 | 224 | 298 | 298 | 258 | 258 |
| 15 | East | Limón | 150 | 154 | 231 | 234 | 292 | 292 | 184 | 196 | 258 | 258 | 197 | 197 | 270 | 270 | 141 | 141 | 268 | 268 | 276 | 284 | 232 | 232 | 274 | 290 | 220 | 232 | 298 | 306 | 258 | 266 |
| 16 | East | Limón | 150 | 154 | 234 | 234 | 296 | 296 | 188 | 188 | 274 | 274 | 201 | 201 | 274 | 274 | 141 | 141 | 268 | 284 | 280 | 284 | 228 | 232 | 274 | 298 | 224 | 232 | 298 | 302 | 254 | 258 |
| 17 | East | Limón | 158 | 158 | 234 | 237 | 288 | 288 | 188 | 188 | 258 | 258 | 0 | 0 | 274 | 282 | 0 | 0 | 276 | 284 | 280 | 288 | 228 | 232 | 290 | 290 | 220 | 240 | 0 | 0 | 258 | 258 |
| 18 | East | Limón | 150 | 150 | 231 | 231 | 292 | 292 | 188 | 188 | 270 | 274 | 189 | 197 | 278 | 278 | 141 | 145 | 268 | 288 | 292 | 292 | 228 | 228 | 290 | 290 | 244 | 248 | 0 | 0 | 254 | 254 |
| 19 | East | Limón | 150 | 154 | 237 | 237 | 296 | 296 | 184 | 204 | 254 | 258 | 197 | 201 | 286 | 286 | 137 | 141 | 272 | 272 | 280 | 280 | 232 | 232 | 298 | 298 | 224 | 232 | 298 | 302 | 262 | 262 |
| 20 | East | Limón | 0 | 0 | 234 | 234 | 0 | 0 | 188 | 188 | 270 | 274 | 189 | 197 | 0 | 0 | 141 | 141 | 260 | 284 | 280 | 284 | 228 | 228 | 290 | 290 | 236 | 236 | 298 | 306 | 258 | 266 |
| 21 | East | Limón | 0 | 0 | 234 | 237 | 304 | 304 | 184 | 184 | 258 | 258 | 201 | 201 | 278 | 278 | 141 | 141 | 276 | 280 | 288 | 288 | 240 | 240 | 274 | 290 | 232 | 232 | 0 | 0 | 266 | 266 |
| 22 | East | Limón | 150 | 158 | 231 | 231 | 296 | 296 | 188 | 204 | 258 | 274 | 189 | 189 | 266 | 286 | 137 | 141 | 268 | 284 | 280 | 292 | 228 | 228 | 290 | 290 | 220 | 232 | 294 | 302 | 258 | 258 |
| 23 | East | Limón | 158 | 158 | 228 | 228 | 292 | 312 | 188 | 196 | 270 | 274 | 185 | 189 | 278 | 278 | 137 | 145 | 268 | 268 | 280 | 284 | 228 | 228 | 290 | 294 | 236 | 236 | 294 | 302 | 258 | 258 |
| 24 | East | Limoncito | 150 | 154 | 228 | 234 | 292 | 292 | 184 | 204 | 258 | 274 | 189 | 197 | 270 | 278 | 133 | 141 | 268 | 268 | 280 | 280 | 228 | 232 | 290 | 290 | 224 | 232 | 298 | 298 | 258 | 258 |
| 25 | East | Limoncito | 0 | 0 | 234 | 237 | 288 | 288 | 184 | 184 | 274 | 274 | 0 | 0 | 274 | 274 | 141 | 141 | 276 | 284 | 280 | 284 | 228 | 228 | 290 | 290 | 232 | 236 | 298 | 298 | 258 | 266 |
| 26 | South East | Puerto Viejo | 150 | 158 | 231 | 231 | 288 | 288 | 188 | 192 | 274 | 274 | 189 | 201 | 270 | 282 | 141 | 141 | 284 | 284 | 280 | 284 | 228 | 228 | 290 | 298 | 220 | 244 | 298 | 306 | 250 | 250 |
| 27 | South East | Puerto Viejo | 150 | 150 | 0 | 0 | 292 | 292 | 184 | 184 | 258 | 270 | 185 | 197 | 270 | 270 | 145 | 145 | 280 | 280 | 0 | 0 | 240 | 240 | 274 | 274 | 228 | 236 | 298 | 302 | 258 | 266 |
| 28 | South East | Puerto Viejo | 150 | 154 | 237 | 237 | 288 | 288 | 184 | 188 | 258 | 270 | 185 | 201 | 274 | 278 | 129 | 133 | 280 | 280 | 276 | 280 | 232 | 232 | 278 | 290 | 236 | 240 | 302 | 302 | 266 | 266 |
| 29 | South East | Puerto Viejo | 154 | 154 | 228 | 228 | 292 | 296 | 184 | 204 | 274 | 274 | 189 | 189 | 274 | 278 | 141 | 141 | 276 | 276 | 284 | 284 | 232 | 236 | 274 | 294 | 240 | 240 | 302 | 302 | 258 | 258 |
| 30 | South East | Bri Bri | 150 | 154 | 228 | 228 | 292 | 300 | 184 | 188 | 258 | 258 | 189 | 189 | 278 | 278 | 141 | 141 | 272 | 276 | 284 | 284 | 236 | 236 | 282 | 298 | 228 | 236 | 298 | 302 | 262 | 266 |
| 31 | South East | Bri Bri | 150 | 154 | 228 | 228 | 292 | 296 | 184 | 184 | 258 | 258 | 189 | 197 | 278 | 282 | 141 | 141 | 272 | 272 | 272 | 276 | 236 | 240 | 282 | 290 | 236 | 236 | 298 | 310 | 258 | 266 |
| 32 | South East | Bri Bri | 150 | 158 | 234 | 237 | 292 | 296 | 204 | 204 | 258 | 278 | 185 | 189 | 274 | 278 | 141 | 145 | 276 | 280 | 280 | 288 | 228 | 240 | 274 | 294 | 228 | 236 | 302 | 306 | 254 | 258 |
| 33 | South East | Cahuita | 150 | 158 | 234 | 237 | 292 | 296 | 188 | 204 | 274 | 278 | 185 | 197 | 274 | 290 | 141 | 149 | 276 | 276 | 280 | 284 | 240 | 240 | 286 | 290 | 232 | 240 | 302 | 306 | 254 | 258 |
| 34 | South East | Cahuita | 150 | 158 | 234 | 234 | 296 | 296 | 184 | 184 | 258 | 274 | 189 | 201 | 270 | 274 | 129 | 141 | 260 | 280 | 276 | 288 | 232 | 240 | 294 | 294 | 228 | 244 | 294 | 306 | 258 | 258 |
| 35 | South East | Cahuita | 150 | 154 | 234 | 237 | 296 | 300 | 188 | 188 | 258 | 278 | 197 | 201 | 274 | 278 | 145 | 145 | 260 | 276 | 280 | 284 | 232 | 240 | 290 | 290 | 236 | 240 | 298 | 302 | 258 | 258 |
| 36 | South East | Cahuita | 154 | 158 | 234 | 234 | 292 | 296 | 184 | 188 | 258 | 258 | 189 | 201 | 270 | 274 | 129 | 141 | 276 | 276 | 284 | 288 | 240 | 240 | 290 | 294 | 228 | 236 | 294 | 298 | 258 | 258 |
| 37 | South East | Cahuita | 150 | 154 | 234 | 237 | 296 | 296 | 188 | 204 | 258 | 274 | 189 | 197 | 278 | 286 | 133 | 141 | 268 | 268 | 276 | 288 | 232 | 240 | 290 | 294 | 236 | 240 | 294 | 302 | 258 | 258 |
| 38 | South East | Cahuita | 150 | 158 | 234 | 234 | 292 | 296 | 184 | 184 | 274 | 278 | 185 | 201 | 274 | 286 | 141 | 141 | 268 | 276 | 280 | 292 | 240 | 240 | 274 | 294 | 236 | 236 | 294 | 302 | 258 | 266 |
| 39 | South East | Cahuita | 150 | 150 | 231 | 237 | 288 | 288 | 188 | 188 | 258 | 258 | 197 | 197 | 274 | 282 | 141 | 145 | 268 | 268 | 272 | 280 | 228 | 232 | 274 | 290 | 220 | 236 | 294 | 298 | 258 | 266 |
| 40 | South East | Cahuita | 0 | 0 | 0 | 0 | 292 | 292 | 184 | 184 | 258 | 278 | 189 | 189 | 274 | 274 | 141 | 141 | 284 | 288 | 284 | 284 | 232 | 232 | 274 | 290 | 236 | 236 | 302 | 302 | 258 | 266 |
| 41 | South East | Cahuita | 150 | 154 | 234 | 237 | 296 | 296 | 184 | 188 | 258 | 278 | 0 | 0 | 0 | 0 | 133 | 133 | 280 | 288 | 272 | 288 | 232 | 240 | 290 | 294 | 236 | 236 | 294 | 302 | 266 | 266 |
| 42 | South East | Cahuita | 150 | 158 | 234 | 234 | 0 | 0 | 188 | 204 | 258 | 270 | 0 | 0 | 0 | 0 | 133 | 145 | 276 | 276 | 276 | 284 | 240 | 244 | 274 | 294 | 240 | 240 | 302 | 306 | 258 | 258 |
| 43 | South East | Cahuita | 0 | 0 | 0 | 0 | 296 | 296 | 184 | 188 | 258 | 274 | 0 | 0 | 0 | 0 | 141 | 141 | 276 | 276 | 276 | 284 | 240 | 240 | 290 | 294 | 228 | 244 | 294 | 294 | 254 | 254 |
| 44 | South East | Cahuita | 150 | 150 | 228 | 234 | 296 | 296 | 284 | 288 | 258 | 278 | 0 | 0 | 274 | 278 | 0 | 0 | 276 | 288 | 272 | 280 | 232 | 232 | 290 | 294 | 236 | 236 | 0 | 0 | 266 | 266 |
| 45 | South East | Cahuita | 150 | 158 | 234 | 234 | 292 | 296 | 188 | 188 | 258 | 258 | 197 | 201 | 270 | 274 | 141 | 145 | 276 | 288 | 280 | 288 | 232 | 240 | 290 | 290 | 240 | 244 | 294 | 298 | 258 | 258 |
| 46 | South East | Cahuita | 150 | 150 | 0 | 0 | 292 | 296 | 184 | 188 | 258 | 258 | 0 | 0 | 278 | 286 | 133 | 141 | 268 | 280 | 0 | 0 | 232 | 240 | 286 | 290 | 236 | 236 | 294 | 298 | 258 | 266 |
| 47 | South East | Cahuita | 0 | 0 | 0 | 0 | 288 | 292 | 188 | 188 | 278 | 278 | 201 | 201 | 274 | 274 | 137 | 145 | 276 | 288 | 0 | 0 | 236 | 240 | 286 | 290 | 236 | 236 | 298 | 298 | 258 | 258 |
| 48 | South East | Cahuita | 154 | 158 | 234 | 237 | 292 | 292 | 184 | 204 | 258 | 274 | 189 | 197 | 278 | 282 | 141 | 145 | 260 | 268 | 280 | 288 | 232 | 232 | 286 | 290 | 236 | 240 | 298 | 302 | 258 | 266 |
| 49 | South East | Cahuita | 150 | 150 | 234 | 237 | 296 | 296 | 192 | 192 | 258 | 278 | 185 | 189 | 278 | 278 | 141 | 145 | 276 | 276 | 280 | 288 | 228 | 228 | 290 | 298 | 220 | 224 | 302 | 302 | 258 | 258 |
| 50 | South East | Cahuita | 150 | 150 | 234 | 234 | 292 | 292 | 188 | 188 | 258 | 274 | 185 | 185 | 278 | 282 | 137 | 141 | 276 | 276 | 280 | 280 | 232 | 240 | 274 | 294 | 236 | 244 | 302 | 302 | 258 | 266 |
| 51 | South East | Cahuita | 158 | 158 | 0 | 0 | 288 | 288 | 184 | 188 | 258 | 270 | 0 | 0 | 286 | 286 | 141 | 141 | 268 | 284 | 280 | 284 | 228 | 232 | 274 | 290 | 224 | 224 | 298 | 302 | 258 | 266 |
| 52 | South East | Cahuita | 150 | 150 | 234 | 237 | 288 | 292 | 184 | 188 | 258 | 258 | 189 | 197 | 274 | 278 | 129 | 129 | 288 | 288 | 280 | 284 | 232 | 240 | 290 | 294 | 224 | 236 | 302 | 306 | 258 | 258 |
| 53 | South East | Cahuita | 150 | 154 | 234 | 234 | 0 | 0 | 184 | 188 | 274 | 274 | 185 | 197 | 278 | 286 | 137 | 145 | 288 | 288 | 0 | 0 | 236 | 240 | 286 | 290 | 240 | 244 | 298 | 302 | 258 | 266 |
| 54 | South East | Cahuita | 150 | 150 | 234 | 237 | 292 | 304 | 188 | 188 | 258 | 258 | 197 | 201 | 278 | 286 | 141 | 141 | 268 | 280 | 280 | 288 | 232 | 240 | 290 | 294 | 240 | 248 | 298 | 298 | 258 | 258 |
| 55 | South East | Cahuita | 150 | 158 | 234 | 237 | 296 | 300 | 184 | 184 | 258 | 274 | 189 | 193 | 278 | 282 | 129 | 137 | 268 | 276 | 280 | 288 | 240 | 240 | 294 | 294 | 236 | 236 | 298 | 302 | 258 | 258 |
| 56 | South East | Cahuita | 150 | 158 | 234 | 237 | 296 | 296 | 184 | 204 | 258 | 274 | 201 | 201 | 282 | 286 | 141 | 141 | 276 | 280 | 284 | 288 | 232 | 236 | 274 | 290 | 236 | 244 | 294 | 302 | 254 | 258 |
| 57 | South East | Penshurt | 0 | 0 | 237 | 237 | 296 | 296 | 204 | 204 | 274 | 274 | 0 | 0 | 278 | 286 | 0 | 0 | 272 | 272 | 276 | 276 | 240 | 240 | 274 | 290 | 232 | 240 | 294 | 298 | 258 | 258 |
| 58 | South East | Penshurt | 150 | 150 | 0 | 0 | 296 | 296 | 188 | 192 | 258 | 274 | 0 | 0 | 270 | 278 | 129 | 137 | 268 | 280 | 0 | 0 | 240 | 240 | 274 | 294 | 224 | 232 | 302 | 302 | 258 | 266 |
| 59 | South East | Penshurt | 150 | 158 | 237 | 237 | 288 | 296 | 204 | 204 | 270 | 274 | 201 | 201 | 278 | 282 | 133 | 141 | 272 | 276 | 276 | 284 | 240 | 244 | 274 | 290 | 232 | 240 | 294 | 298 | 258 | 258 |
| 60 | South East | Aviarios | 150 | 150 | 234 | 237 | 296 | 296 | 188 | 188 | 258 | 274 | 185 | 185 | 278 | 286 | 141 | 145 | 276 | 284 | 276 | 284 | 232 | 232 | 274 | 294 | 236 | 244 | 302 | 302 | 258 | 266 |
| 61 | South East | Aviarios | 150 | 158 | 234 | 237 | 292 | 292 | 188 | 188 | 258 | 258 | 189 | 193 | 278 | 286 | 141 | 141 | 268 | 276 | 280 | 284 | 240 | 240 | 286 | 294 | 236 | 240 | 294 | 302 | 258 | 266 |
| 62 | South East | Aviarios | 150 | 158 | 237 | 237 | 288 | 296 | 188 | 188 | 258 | 274 | 185 | 189 | 270 | 274 | 141 | 141 | 280 | 284 | 280 | 288 | 228 | 232 | 294 | 294 | 240 | 240 | 302 | 302 | 254 | 258 |
| 63 | South East | Aviarios | 0 | 0 | 228 | 234 | 292 | 292 | 188 | 188 | 254 | 258 | 0 | 0 | 274 | 274 | 133 | 137 | 268 | 280 | 280 | 284 | 232 | 240 | 274 | 290 | 240 | 240 | 298 | 302 | 258 | 258 |
| 64 | South East | Aviarios | 150 | 158 | 234 | 237 | 288 | 300 | 188 | 188 | 258 | 274 | 189 | 201 | 270 | 278 | 141 | 145 | 272 | 284 | 280 | 280 | 232 | 240 | 290 | 294 | 236 | 240 | 294 | 302 | 258 | 258 |
| 65 | South East | Aviarios | 0 | 0 | 234 | 237 | 292 | 296 | 188 | 188 | 258 | 258 | 201 | 201 | 274 | 278 | 0 | 0 | 284 | 284 | 280 | 284 | 236 | 240 | 290 | 290 | 232 | 236 | 290 | 298 | 258 | 266 |
| 66 | South East | Río Banano | 150 | 150 | 234 | 237 | 0 | 0 | 188 | 188 | 274 | 274 | 189 | 201 | 270 | 274 | 141 | 145 | 268 | 280 | 280 | 280 | 228 | 232 | 274 | 290 | 236 | 240 | 302 | 302 | 254 | 258 |
| 67 | South East | Río Banano | 154 | 158 | 234 | 234 | 288 | 288 | 184 | 188 | 254 | 254 | 185 | 201 | 274 | 278 | 133 | 133 | 276 | 276 | 276 | 280 | 232 | 240 | 274 | 294 | 232 | 232 | 294 | 302 | 254 | 258 |
| 68 | South East | Río Banano | 150 | 150 | 237 | 237 | 296 | 296 | 188 | 188 | 258 | 270 | 189 | 189 | 266 | 274 | 137 | 145 | 268 | 268 | 288 | 288 | 244 | 244 | 290 | 298 | 220 | 236 | 0 | 0 | 258 | 258 |
| 69 | West | San José | 154 | 158 | 234 | 237 | 0 | 0 | 188 | 188 | 258 | 270 | 185 | 201 | 274 | 274 | 137 | 137 | 276 | 284 | 280 | 280 | 232 | 240 | 274 | 274 | 224 | 232 | 302 | 302 | 258 | 266 |
| 70 | West | San José | 0 | 0 | 231 | 231 | 296 | 296 | 188 | 204 | 274 | 274 | 0 | 0 | 0 | 0 | 145 | 145 | 268 | 268 | 0 | 0 | 228 | 228 | 286 | 286 | 220 | 220 | 298 | 298 | 250 | 250 |
| 71 | West | San José | 150 | 158 | 0 | 0 | 296 | 304 | 204 | 204 | 274 | 274 | 189 | 197 | 270 | 278 | 141 | 141 | 268 | 280 | 280 | 284 | 228 | 228 | 290 | 290 | 220 | 228 | 298 | 302 | 250 | 258 |
| 72 | West | San José | 150 | 150 | 231 | 237 | 304 | 304 | 188 | 196 | 274 | 274 | 197 | 197 | 278 | 286 | 145 | 145 | 268 | 268 | 280 | 288 | 228 | 228 | 278 | 290 | 220 | 236 | 294 | 298 | 258 | 258 |
| 73 | West | San José | 150 | 154 | 234 | 234 | 292 | 300 | 188 | 188 | 270 | 270 | 197 | 197 | 266 | 266 | 141 | 141 | 268 | 268 | 0 | 0 | 240 | 240 | 290 | 290 | 236 | 236 | 302 | 310 | 258 | 274 |
| 74 | West | San José | 0 | 0 | 231 | 231 | 288 | 296 | 188 | 204 | 270 | 274 | 189 | 201 | 274 | 278 | 141 | 141 | 268 | 288 | 0 | 0 | 0 | 0 | 290 | 294 | 236 | 244 | 0 | 0 | 258 | 258 |
| 75 | West | San José | 150 | 150 | 231 | 231 | 292 | 304 | 188 | 188 | 270 | 274 | 189 | 189 | 270 | 282 | 141 | 145 | 268 | 268 | 280 | 284 | 228 | 228 | 290 | 290 | 236 | 236 | 298 | 298 | 258 | 266 |
| 76 | West | San José | 150 | 150 | 231 | 231 | 292 | 292 | 196 | 204 | 270 | 270 | 189 | 189 | 278 | 282 | 141 | 145 | 268 | 276 | 280 | 284 | 228 | 244 | 290 | 294 | 220 | 220 | 298 | 298 | 250 | 258 |
| 77 | West | San José | 150 | 150 | 234 | 234 | 288 | 288 | 188 | 188 | 270 | 270 | 197 | 201 | 270 | 286 | 141 | 141 | 260 | 260 | 280 | 280 | 240 | 240 | 298 | 298 | 220 | 236 | 294 | 298 | 254 | 266 |
| 78 | West | San José | 150 | 150 | 228 | 234 | 288 | 292 | 184 | 184 | 258 | 258 | 197 | 197 | 274 | 278 | 141 | 141 | 268 | 268 | 276 | 288 | 236 | 236 | 274 | 290 | 240 | 240 | 298 | 298 | 258 | 258 |
| 79 | North | Turrialba | 150 | 158 | 237 | 237 | 296 | 300 | 188 | 188 | 270 | 270 | 189 | 189 | 274 | 278 | 141 | 145 | 268 | 284 | 280 | 280 | 228 | 232 | 290 | 298 | 220 | 240 | 294 | 298 | 250 | 258 |
| 80 | North | Turrialba | 150 | 150 | 231 | 231 | 296 | 296 | 188 | 196 | 266 | 270 | 189 | 189 | 274 | 278 | 145 | 145 | 264 | 264 | 280 | 280 | 228 | 240 | 274 | 290 | 220 | 232 | 294 | 306 | 250 | 258 |
| 81 | North | Siquirres | 150 | 150 | 231 | 237 | 292 | 296 | 196 | 204 | 270 | 270 | 197 | 201 | 274 | 286 | 141 | 145 | 276 | 288 | 280 | 292 | 228 | 240 | 290 | 290 | 236 | 244 | 298 | 298 | 250 | 258 |
| 82 | North | Siquirres | 150 | 150 | 237 | 237 | 292 | 292 | 188 | 188 | 270 | 270 | 197 | 201 | 286 | 286 | 141 | 141 | 264 | 284 | 288 | 288 | 228 | 236 | 294 | 294 | 240 | 240 | 302 | 302 | 250 | 250 |
| 83 | North | Siquirres | 150 | 158 | 231 | 237 | 288 | 296 | 184 | 188 | 270 | 274 | 197 | 201 | 278 | 286 | 137 | 141 | 260 | 284 | 276 | 280 | 228 | 244 | 290 | 294 | 236 | 240 | 290 | 302 | 254 | 258 |
| 84 | North | Siquirres | 0 | 0 | 231 | 237 | 288 | 288 | 188 | 204 | 270 | 274 | 201 | 201 | 274 | 278 | 141 | 141 | 276 | 288 | 280 | 288 | 228 | 236 | 290 | 294 | 220 | 240 | 298 | 302 | 258 | 258 |
| 85 | North | Guacimo | 150 | 150 | 0 | 0 | 288 | 292 | 188 | 188 | 274 | 274 | 189 | 189 | 270 | 270 | 141 | 141 | 276 | 276 | 280 | 288 | 228 | 244 | 290 | 290 | 240 | 244 | 298 | 298 | 258 | 258 |
| 86 | North | Guacimo | 0 | 0 | 237 | 237 | 292 | 296 | 188 | 196 | 274 | 274 | 189 | 189 | 274 | 282 | 141 | 145 | 272 | 276 | 280 | 292 | 228 | 240 | 290 | 294 | 220 | 240 | 298 | 306 | 250 | 250 |
| 87 | North | Guacimo | 0 | 0 | 231 | 231 | 296 | 296 | 184 | 188 | 270 | 270 | 189 | 189 | 274 | 278 | 0 | 0 | 260 | 260 | 292 | 292 | 228 | 228 | 294 | 298 | 240 | 240 | 298 | 298 | 254 | 258 |
| 88 | North | Guacimo | 150 | 150 | 228 | 237 | 296 | 304 | 200 | 204 | 270 | 270 | 189 | 189 | 270 | 278 | 137 | 141 | 268 | 288 | 288 | 300 | 228 | 228 | 290 | 294 | 220 | 244 | 302 | 306 | 254 | 258 |
| 89 | North | Guapiles | 150 | 150 | 228 | 231 | 288 | 288 | 188 | 204 | 274 | 274 | 189 | 197 | 266 | 282 | 141 | 145 | 268 | 288 | 284 | 288 | 228 | 228 | 290 | 290 | 232 | 244 | 302 | 302 | 250 | 258 |
| 90 | North | Guapiles | 150 | 158 | 228 | 231 | 288 | 288 | 196 | 204 | 270 | 274 | 189 | 189 | 274 | 278 | 141 | 141 | 268 | 268 | 280 | 292 | 228 | 244 | 290 | 298 | 220 | 240 | 298 | 298 | 250 | 250 |
| 91 | North | Guapiles | 150 | 150 | 0 | 0 | 288 | 288 | 188 | 196 | 270 | 274 | 185 | 189 | 278 | 278 | 141 | 141 | 276 | 284 | 0 | 0 | 228 | 244 | 290 | 290 | 220 | 240 | 298 | 298 | 250 | 258 |
| 92 | North | Guapiles | 150 | 158 | 228 | 231 | 288 | 288 | 188 | 192 | 270 | 274 | 189 | 189 | 270 | 274 | 141 | 141 | 284 | 288 | 284 | 288 | 228 | 228 | 290 | 290 | 220 | 220 | 298 | 302 | 254 | 258 |
| 93 | North | Guapiles | 150 | 158 | 237 | 237 | 292 | 292 | 188 | 204 | 270 | 270 | 189 | 201 | 286 | 286 | 141 | 145 | 264 | 288 | 280 | 288 | 228 | 236 | 290 | 294 | 220 | 240 | 302 | 302 | 250 | 258 |
| 94 | North | Guapiles | 150 | 150 | 228 | 231 | 288 | 288 | 184 | 188 | 270 | 270 | 189 | 189 | 266 | 274 | 145 | 145 | 268 | 276 | 280 | 288 | 228 | 228 | 290 | 290 | 232 | 232 | 0 | 0 | 258 | 258 |
| 95 | North | Guapiles | 150 | 150 | 231 | 231 | 288 | 288 | 188 | 196 | 270 | 274 | 189 | 189 | 278 | 282 | 0 | 0 | 288 | 288 | 288 | 292 | 240 | 240 | 290 | 298 | 232 | 240 | 0 | 0 | 250 | 254 |
| 96 | North | Guapiles | 150 | 158 | 228 | 237 | 292 | 292 | 188 | 204 | 270 | 274 | 189 | 189 | 266 | 282 | 141 | 145 | 260 | 268 | 280 | 288 | 228 | 232 | 290 | 290 | 220 | 240 | 298 | 306 | 254 | 258 |
| 97 | North | Guapiles | 150 | 158 | 231 | 231 | 292 | 308 | 188 | 196 | 270 | 270 | 189 | 189 | 274 | 278 | 141 | 141 | 268 | 276 | 280 | 288 | 228 | 232 | 290 | 298 | 220 | 220 | 298 | 302 | 250 | 250 |
| 98 | North | Guapiles | 0 | 0 | 231 | 231 | 288 | 296 | 184 | 184 | 270 | 274 | 0 | 0 | 278 | 278 | 141 | 145 | 264 | 284 | 280 | 292 | 236 | 236 | 290 | 294 | 220 | 220 | 302 | 302 | 250 | 254 |

**References**

Carlson, T. N., & Sanchez-Azofeifa, G. A. (1999). Satellite Remote Sensing of Land Use Changes in and around San José, Costa Rica. *Remote Sensing of Environment*, *70*, 247–256.

Fagan, M. E., DeFries, R. S., Sesnie, S. E., Arroyo, J. P., Walker, W., Soto, C., … Sanchun, A. (2013). Land cover dynamics following a deforestation ban in northern Costa Rica. *Environmental Research Letters*, *8*, 034017.

Jadin, I., Meyfroidt, P., & Lambin, E. F. (2016). International trade, and land use intensification and spatial reorganization explain Costa Rica’s forest transition. *Environmental Research Letters*, *11*, 035005.

Öborn, I., Bengtsson, J., Hedenus, F., Rydhmer, L., Stenström, M., Vrede, K., … Magnusson, U. (2013). Scenario Development as a Basis for Formulating a Research Program on Future Agriculture: A Methodological Approach. *Ambio*, *42*, 823–839.

Pontius, R. G., Cornell, J. D., & Hall, C. A. S. (2001). Modeling the spatial pattern of land-use change with GEOMOD2: Application and validation for Costa Rica. *Agriculture, Ecosystems & Environment*, *85*, 191–203.

Van Laake, P. E., & Sánchez-Azofeifa, G. A. (2004). Focus on deforestation: Zooming in on hot spots in highly fragmented ecosystems in Costa Rica. *Agriculture, Ecosystems & Environment*, *102*, 3–15.
